# Fabrication of Biogenic Titanium Nanoparticles and Investigating Their Biological Properties for Dental Applications

**DOI:** 10.7759/cureus.44209

**Published:** 2023-08-27

**Authors:** Shubhasri A.S., Tina Sara Biju, Arul Prakash Francis, Gayathri R, Vishnu Priya Veeraraghavan, Kavitha Sankaran

**Affiliations:** 1 Centre of Molecular Medicine and Diagnostics (COMMAND) Department of Biochemistry, Saveetha Dental College and Hospitals, Saveetha Institute of Medical and Technical Sciences, Chennai, IND

**Keywords:** anti-inflammatory, curcumin, myristica fragrans, titanium oxide nanoparticles, green synthesis

## Abstract

Introduction: Oral inflammation, often triggered by infections, injuries, or immune responses, can compromise treatment outcomes, delay healing, and contribute to patient discomfort. The development of green nanoparticle synthesis methods is receiving attention due to their potential advantages over existing approaches. These procedures use commonly available, affordable, and environmentally friendly natural plant extracts. Due to their numerous uses in various industries, titanium oxide nanoparticles (TiO_2_NPs) have attracted the most attention among the nanoparticles. In this study, we present the green synthesis of *Myristica fragrans* (mace) extract as a reductant and stabilizer for the production of curcumin-functionalized TiO_2_NPs (CTN). We additionally evaluated the effectiveness of these nanoparticles as anti-inflammatory agents.

Objective: In this study, we aim to develop biogenic TiO_2_NPs using *Myristica fragrans* as a natural capping agent and functionalized with curcumin for effectively managing oral inflammation in dental applications.

Methods: The nanoparticles were synthesized using the green synthesis method and characterized using various characterization techniques. Biocompatibility was evaluated using hemolytic assays, and the bioactivity of the nanoparticles was assessed using anti-inflammatory assays.

Results: Curcumin-coated M-TiO_2_NPs (MCTN) were successfully synthesized and characterized by various techniques, confirming their morphology, crystallinity, functionalization, elemental composition, size, and stability. *In vitro* bioactivity studies revealed that MCTN exhibited significant anti-inflammatory activity, as evidenced by the inhibition of protein denaturation with minimal hemolytic potential. These findings highlight the potential of MCTN as a promising candidate for anti-inflammatory applications.

Conclusion: Our results suggest that MCTN exhibits promising anti-inflammatory and anti-hemolytic properties. However, further in-depth *in vivo* analysis is required to fully understand their efficacy and toxicity.

## Introduction

In dental therapeutics, the effective management of oral inflammation stands as a crucial cornerstone [1]. This imperative undertaking involves the careful control and mitigation of inflammatory processes within the oral cavity, directly impacting the success of diverse dental treatments. Originating from various sources such as infections, trauma, or immune responses, oral inflammation poses a significant challenge to optimal treatment outcomes, potentially leading to treatment delays, patient discomfort, and compromised healing [2]. Consequently, strategic interventions aimed at addressing oral inflammation not only facilitate an ideal environment for dental procedures but also hold the potential to enhance patient recovery and bolster the overall longevity of oral health.

Nanobiotechnology has gained a lot of attention in recent years for its small size, surface, interface, and quantum effect, as well as its unique physical and chemical properties [3]. It has numerous applications in biomedical applications and engineering (dye degradation, clearing environmental contamination, etc.). Synthesis of nanoparticles can be done by using techniques such as physical (laser ablations, etching), chemical (co-precipitation, hydrothermal synthesis, sol-gel), and green (plant or microbial source) synthesis methods [4]. Out of these, the green synthesis method employs a safe, cost-effective, and environment-friendly approach [5]. TiO_2_NPs have various applications in implantology [6], optical science, biomedical engineering, electronics, etc. In biomedical applications, it is used to coat artificial joints, dental applications, photocatalytic reactions, cosmetics, food, cancer treatment, etc. [7].

*Myristica fragrans*, commonly called "Nutmeg," is widely used as a spice on South Asian continents. In Ayurvedic medicine, this nutmeg is used to treat muscle spasms, rheumatoid arthritis, digestive disorders, etc. [8]. A study showed that lignan, a phytocompound from mace, the seed coat of nutmeg, has hepatoprotective activity [9]. Another component in mace, myristicin, has been shown to have an anti-inflammatory effect in carrageenin-induced edema in rats [10,11]. Various studies have been conducted on the potential antimicrobial activity of *M. fragrans* and were found to be effective against endodontic microorganisms [12,13].

Curcumin, a polyphenol present in the roots of Curcuma longa, has been used as a coloring and flavoring agent for centuries in South Asian countries. It is widely used for its potential health benefits, such as anti-inflammatory, antioxidant, antiviral, anti-arthritic, etc. [14-16]. The major limitation of using curcumin is its poor bioavailability, which leads to rapid metabolism and excretion from the body [17]. It is shown that coating curcumin onto the surface of nanoparticles can improve its efficacy, increase its solubility, and decrease the time of degradation [18,19].

Thus, in this study, we have employed the synthesis of titanium oxide nanoparticles (M-TiO_2_NPs) using *M. fragrans* seed coat extract as a capping agent and functionalized them with curcumin, resulting in curcumin-coated M-TiO_2_NPs (MCTN). These nanoparticles are then characterized using various characterization techniques for confirmation of the material and its morphology [20]. The biocompatibility study was done by performing the hemolytic assay, and its bioactivity was evaluated using the anti-inflammatory assay. This study shows only the preliminary analysis of MCTN; further extensive analysis needs to be done to evaluate its efficacy and toxicity.

## Materials and methods

Sample collection

*M. fragrans* seed coat extract was used for the NP preparation. The seed coats were purchased from a local market in Chennai, India, and authenticated by a botanist.

Chemicals and reagents

Titanium tetra isopropoxide, antibiotic disc, and curcumin were procured from SRL. All additional reagents used in the study were of analytical quality, and MilliQ water was utilized throughout.

Preparation of the extract

To remove residual contaminants from the seed coat of the collected *M. fragrans*, the seed coat was washed repeatedly with distilled water. The seed coat was allowed to dry at room temperature before being crushed into a coarse powder. Two grams of coarse powder was added to 50 mL of MilliQ water heated at 80 °C for 30 minutes. The extract was then collected, filtered using Whatman filter paper, and used for the further synthesis of M-TiO_2_ NPs.

Preparation of M-TiO2 NPs

Three milliliters of titanium tetra-isopropoxide is added to the ethanol-seed coat extract mixture under stirring. The precipitate form is further stirred for two hours at 60 °C. The precipitate is then separated by centrifugation and washed with acetone. Then the precipitate is dried at 70 °C overnight.

Functionalization of M-TiO_2_ NPs with curcumin

About 20 mL of aqueous PEG 4000 (0.05%) solution was added to the 200 mg of M-TiO_2_NPs and stirred using a magnetic stirrer for one hour at 37 °C. The curcumin solution (20 mg/mL in DMSO) was then added and stirred for 30 minutes. The precipitate was then centrifuged for 10 minutes at 8,000 rpm to collect the curcumin-coated M-TiO_2_ NPs (MCTN). This precipitate was subjected to freeze drying and used for further characterization and biocompatibility studies.

Characterization of nanoparticles

M-TiO_2_ NPs and MCTN were re-dispersed in deionized water and characterized using various techniques. The UV-visible spectra of MCTN and M-TiO_2_ NPs were measured with a Jasco UV-visible spectrophotometer at wavelengths ranging from 200 to 800 nm. The functional groups of extract and curcumin present in MCTN were determined using an FTIR spectrum obtained using a Bruker IR spectrometer in the range 4000-500 cm^−1^ in ATR mode. The X-ray diffraction (XRD) pattern of MCTN was measured in the region of 20-80° using an X-ray diffractometer of characteristic Cu-K radiation (= 1.5406) at a scan rate of 0.05°/min and a time constant of two seconds. Scanning electron microscopy (JEOL JSM-IT800 SEM, Japan) was used to analyze surface morphology, and energy dispersive X-ray (EDAX) analysis was performed utilizing the JSM-IT800 SEM apparatus with a silicon drift detector (Oxford X-MaxN 50 mm^2^, Oxford apparatus, United Kingdom).

In-vitro anti-inflammatory activity

The anti-inflammatory activity of MCTN was assessed by its ability to inhibit the denaturation of bovine serum albumin, as demonstrated by Pandiyan et al. [21] with minor modifications. PBS was used to dilute different amounts of NPs (25, 50, 100, and 200 µg/mL). One milliliter of 1% BSA was added to this and incubated for 20 minutes at 37 °C and 10 minutes at 75 °C. After it cooled down, the absorbance at 660 nm was measured using pure water as a blank [22-24]. The findings were obtained using the formula below:

Percentage Inhibition = [(ControlAbs − SampleAbs)/ControlAbs] × 100

Hemolytic assay

A hemocompatibility assay evaluated the interaction between erythrocytes and MCTN. This study adhered to prior publications' methods [25]. EDTA-treated whole human blood was ethically collected from patients in vacutainers and centrifuged at 1500 × *g* for five minutes to isolate erythrocytes (RBCs) by separating plasma. These RBCs were washed three times with phosphate-buffered saline (pH 7.4) and subsequently diluted to 10% of their original concentration. Samples of 200 𝜇L erythrocyte suspension (12.5, 25, 50, 100, and 200 𝜇g/mL) were combined, reaching a final volume of 1 mL using PBS. The mixture was incubated at 37 °C for an hour, followed by centrifugation at 1500 × *g* for five minutes. The supernatant was transferred to 96-well plates, and absorbance at 540 nm was measured with an ELISA plate reader. Negative controls were PBS-treated cells, while positive controls were deionized water-treated cells. This procedure was replicated three times, and the hemolysis percentage was calculated using the below formula [26]:

% Hemolysis = [(TestAbs − BlankAbs)/(ControlAbs − BlankAbs)] × 100

Statistical analysis

Statistical analyses were conducted with GraphPad Prism 6.0 software (GraphPad Software, Inc., La Jolla, CA). Data were expressed as mean ± SD (n = 3). Data from different time points were compared using a one-way ANOVA followed by the Students-Newman-Keuls test. The criterion for statistical significance for all tests was set at P < 0.05, and levels of significance were represented for each result.

## Results

In this current study, the M-TiO_2_NPs were synthesized using *M. fragrans* as a capping agent and functionalized with curcumin. The nanoparticles are then characterized using various characterization techniques. The biocompatibility study was done by performing the hemolytic assay, and its bioactivity was evaluated using the anti-inflammatory.

Characterization of M-TiO_2 _NPs

**Figure 1:**
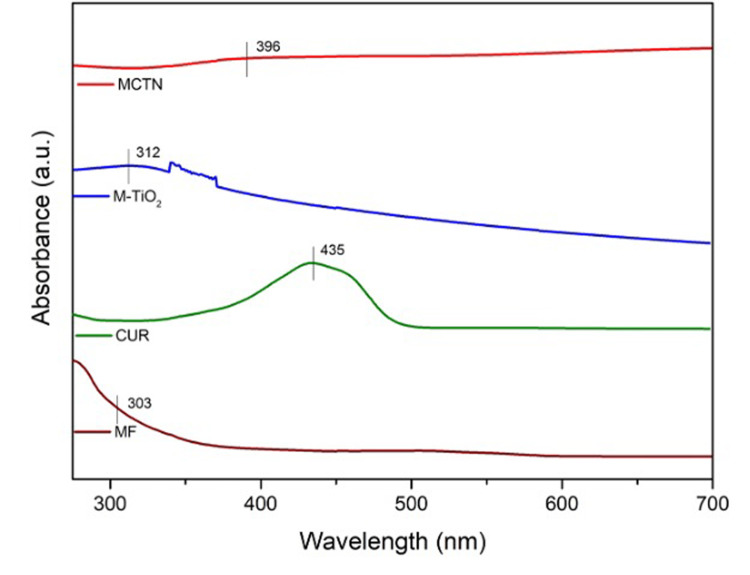
UV spectra of MF, Cur, M-TiO2NPs, and MCTN. MF: *Myristica fragrans*, Cur: curcumin, M-TiO_2_NPs: *M. fragrans-*based titanium nanoparticles, MCTN: Curcumin-coated M-TiO_2_NPs.

The UV-visible spectral analysis for M-TiO_2_NPs and MCTN showed a typical surface plasmon resonance (SPR) peak with maximum absorbance at 312 nm and 396 nm, respectively. Figure 1 represents the UV spectra of MCTN. The UV-visible spectral analysis for MCTN showed a typical SPR peak with maximum absorbance at 396 nm, confirming the MCTN formation, which is evidenced by the shift in maximum absorbance compared to the extract.

**Figure 2:**
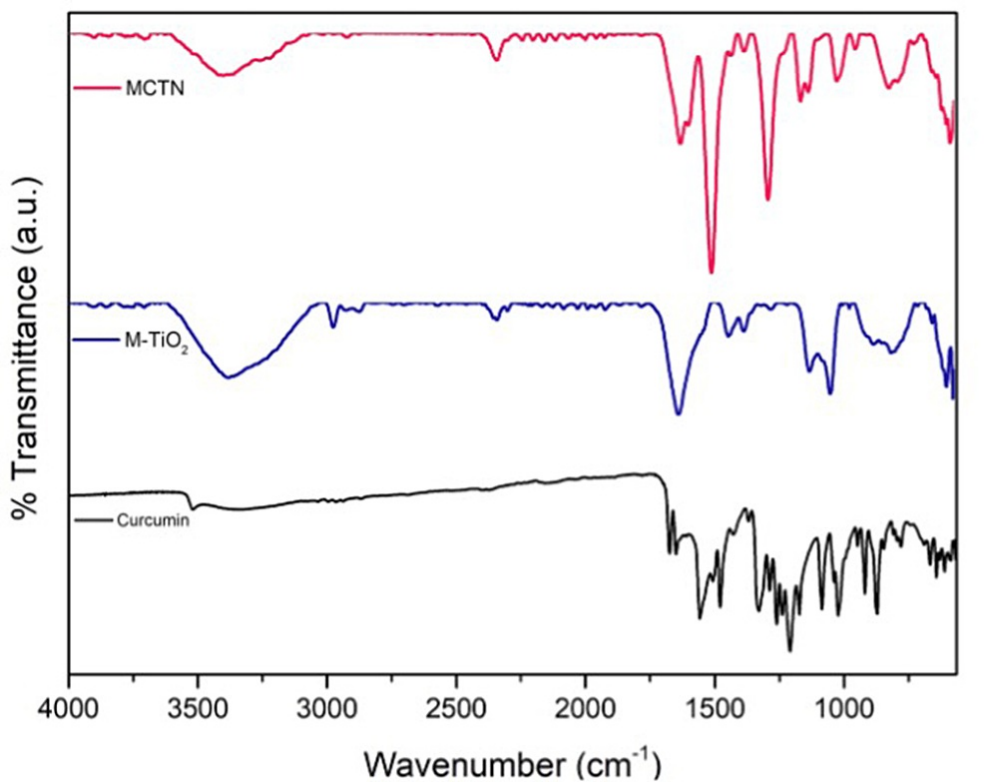
FT-IR spectrum of Cur, M-TiO2NPs, and MCTN. Cur: curcumin, M-TiO_2_NPs: *M. fragrans-*based titanium nanoparticles, MCTN: curcumin-coated M-TiO_2_NPs.

FT-IR spectra were recorded between 4000 and 500 cm^−1^. Figure 2 represents the result of the M-TiO_2_ NPs and the synthesized MCTN. The FT-IR spectra of M-TiO_2_ NPs showed strong absorption bands at 3379, 2973, 2338, 1631, 1441, 1126, 1045, and 570 cm^−1^. The characteristic stretching at 3379 cm^−1^ confirms the OH group present in the extract used for M-TiO_2_ NP formation, which is evidenced by the shift in maximum absorbance compared to the extract. The FT-IR spectrum of MCTN showed strong absorption bands at 3397, 2338, 1624, 1504, 1285, 1158, 1018, and 578 cm^−1^. The characteristic stretching at 3397 cm^−1^ confirms the presence of the OH group in the extract used for MCTN formation, which is evidenced by the shift in maximum absorbance compared to the extract.

**Figure 3:**
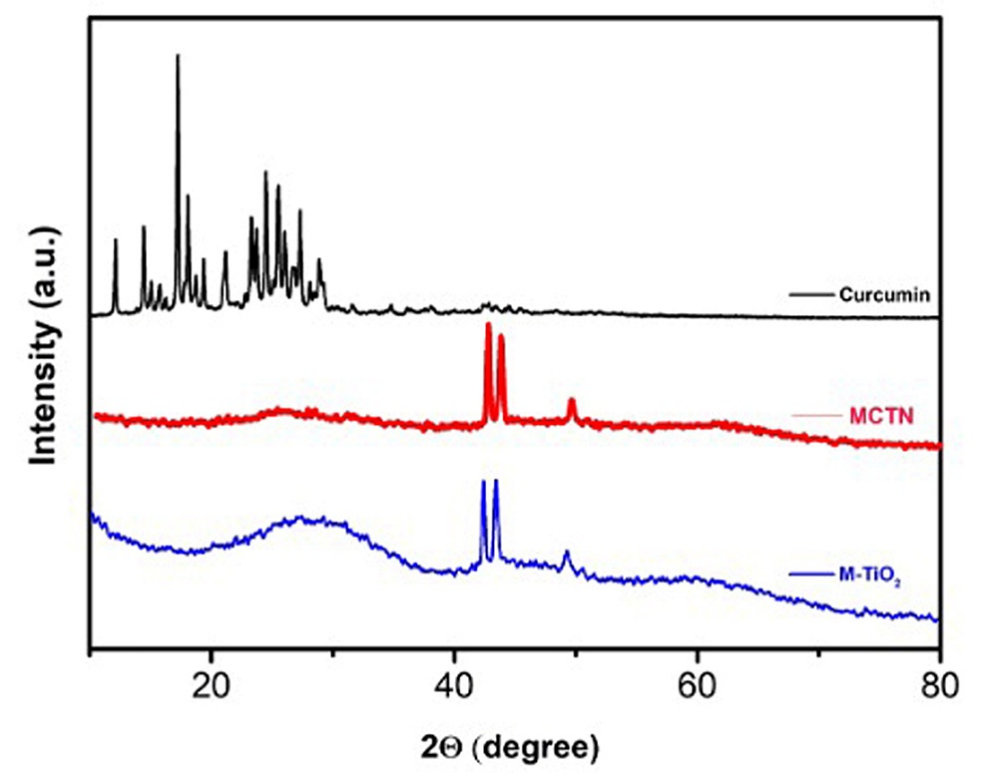
XRD pattern of Cur, M-TiO2 NPs, and MCTN. Cur: curcumin, M-TiO_2_NPs: *M. fragrans-*based titanium nanoparticles, MCTN: curcumin-coated M-TiO_2_NPs.

Figure 3 represents the XRD pattern of Cur, M-TiO_2_ NPs, and MCTN and displays the amorphous nature of the M-TiO_2_ NPs. The positions of the peaks were compared to the JCPDF database, which revealed that the sample has an amorphous structure consistent with titanium oxide. The peak observed at 10° indicates the successful coating of curcumin on M-TiO_2_ NPs.

**Figure 4:**
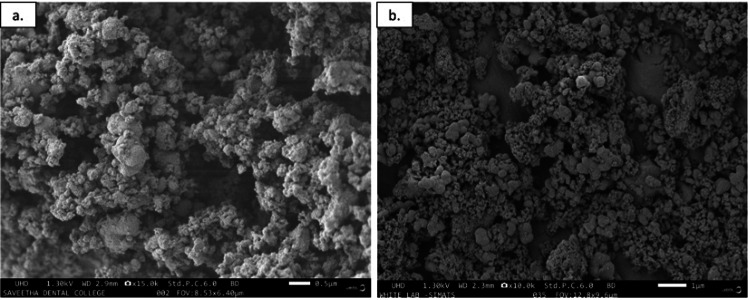
SEM micrograph of (a) M-TiO2NPs and (b) MCTN. SEM: scanning electron microscope, M-TiO_2_NPs: *M. fragrans-*based titanium nanoparticles, MCTN: curcumin-coated M-TiO_2_NPs.

**Figure 5:**
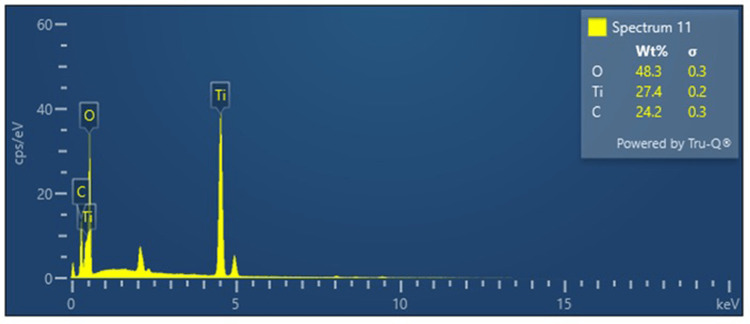
EDAX spectrum of MCTN. EDAX: energy dispersive X-ray analysis, MCTN: curcumin-coated M-TiO_2_NPs.

The morphology of the synthesized nanoparticles was determined by scanning electron microscopy. M-TiO_2_ NPs exhibit agglomerated, spherical-shaped particles with a size range of around 80 nm. On the other hand, the coating of curcumin increases the particle size to 100 nm. Figure 4 represents the morphology of M-TiO_2_ NPs and MCTN. EDAX of wt% of green synthesized M-TiO_2_NPs was found to be greater at 27.4%. This result confirms that M-TiO_2_ NPs were present. Figure 5 represents the EDAX of synthesized MCTN.

In vitroanti-inflammatory activity

**Figure 6:**
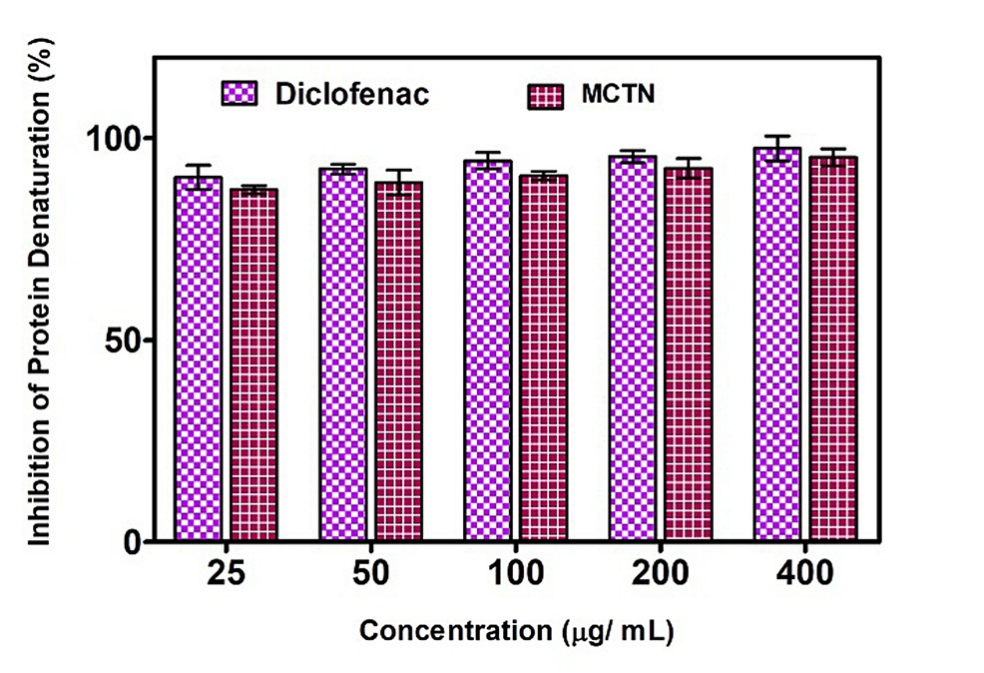
Anti-inflammatory activity of MCTN. MCTN: curcumin-coated M-TiO_2_NPs. The experiment was performed in triplicates and values are expressed in mean ± SD.

The in vitro anti-inflammatory assay results showed that MCTN created using *M. fragrans* (Mace) extract substantially and dose-dependently reduced the denaturation of BSA (Figure 6). This suggests that the nanoparticles have the potential to have an anti-inflammatory effect and could be applied to the creation of new anti-inflammatory drugs.

Hemolytic assay

**Figure 7:**
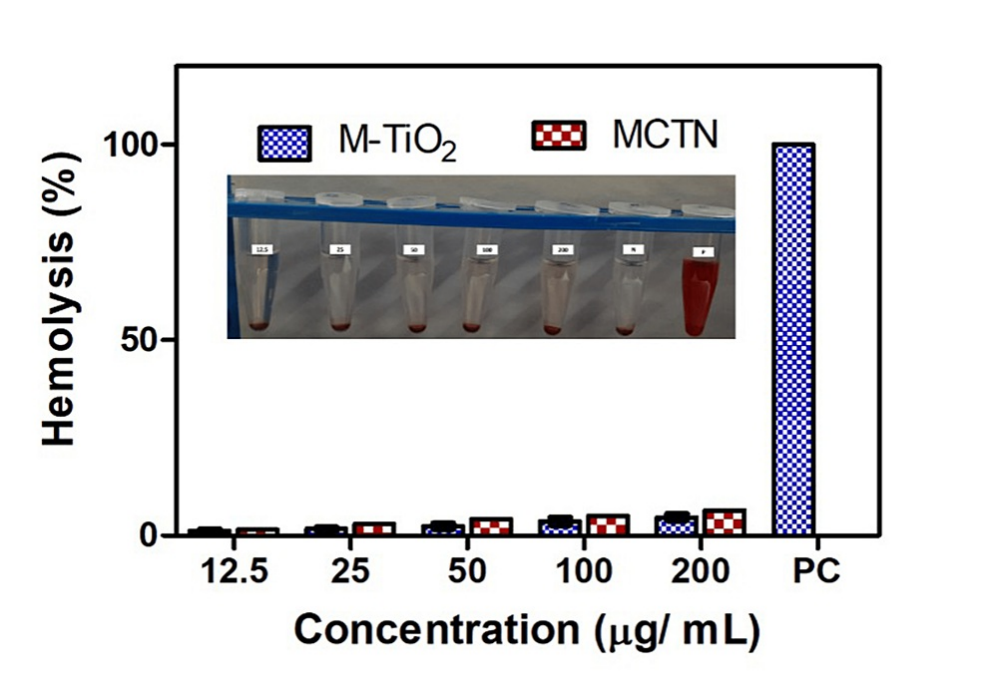
Hemolytic activity of M-TiO2NPs and MCTN. M-TiO_2_NPs: *M. fragrans-*based titanium nanoparticles, MCTN: curcumin-coated M-TiO_2_NPs. The experiment was performed in triplicates and values are expressed in mean ± SD. *Values are statistically significant from the control group (P<0.05).

MCTN showed less than 5% hemolysis in erythrocytes at various concentrations of 10, 25, 50, 100, and 200 μg/mL in comparison with the control. Figures 5-6 represent the hemolytic assay test results of M-TiO_2_NPs and MCTN (Figure 7).

## Discussion

The absorption pattern of MCTN that was made using a seed coat extract of *M. fragrans* (mace) and curcumin was examined using UV-Vis spectrometry. The surface plasmon resonance of the nanoparticles is responsible for the absorption peak at 396 nm, which correlates with a previous study by Abdalla et al. [27]. It was discovered that the peak's strength increased as the nanoparticle concentration increased, indicating the creation of stable, homogenous nanoparticles. The outcomes show that MCTN was successfully synthesized in a green and sustainable manner.

The FT-IR spectrum of M-TiO_2_NPs showed strong absorption bands at 3379, 2973, 2338, 1631, 1441, 1126, 1045, and 570 cm^−1^, which correlated with a previous study [28]. The characteristic peak at 3379 cm^−1^ corresponds to medium N-H stretching of an aliphatic primary amine. The peak at 2973 cm^−1^ corresponds to medium C-H stretching of alkane. The peak at 2338 cm^−1^ corresponds to strong O=C=O stretching of carbon dioxide. The peak at 1631 cm^−​​​​​​​1^ corresponds to medium C=C stretching of disubstituted alkene. The peak at 1441 cm^−​​​​​​​1^ corresponds to the medium O-H bending of carboxylic acid. The peak at 1126 cm^−​​​​​​​1^ corresponds to strong C-O stretching of a secondary alcohol. The peak at 1045 cm^−​​​​​​​1^ corresponds to strong, broad CO-O-CO stretching of anhydride.

Similarly, the FT-IR spectrum of MCTN showed strong absorption bands at 3397, 2338, 1624, 1504, 1285, 1158, 1018, and 578 cm^−​​​​​​​1^. The characteristic peak at 3397 cm^−​​​​​​​1^ corresponds to medium N-H stretching of an aliphatic primary amine. The peak at 1624 cm^−​​​​​​​1^ corresponds to medium C=C stretching of conjugated alkene. The peak at 1504 cm^−​​​​​​​1^ corresponds to strong N-O stretching of the nitro compound. The peak at 1285 cm^−​​​​​​​1^ corresponds to the strong C-O stretching of an aromatic ester. The peak at 1158 cm-1 corresponds to strong C-O stretching of aliphatic ether. The peak at 1018 cm-1 corresponds to medium C-N stretching of amine.

The crystalline structure of the synthesized MCTN was determined using XRD research. The MCTN XRD pattern revealed prominent diffraction peaks at 28.3°, 37.8°, 48.1°, 54.2°, 55.2°, 62.7°, and 68.9°, which corresponded to the anatase M-TiO_2_ NPs crystal planes (101), (004), (200), (105), (211), (204), and (215), respectively, which correlated with the previous report by Ricci et al. [29]. Pure anatase M-TiO_2_ NP nanoparticles were formed because no other phases' diffraction peaks could be seen.

The MCTN was spherical and had a fairly consistent size distribution, as evidenced by the SEM pictures, which are consistent with findings from previous reports [30]. It was found that the typical particle size was around 100 nm. The nanoparticles' stable nature and appropriateness for a variety of applications were shown by their good dispersibility and good separation from one another. The SEM results show that M-TiO_2_ NPs and MCTN were successfully synthesized employing *M. fragrans* extract as a green reducing and stabilizing agent. The presence of titanium, oxygen, carbon, and traces of curcumin in the EDX analysis of the synthesized MCTN indicated successful functionalization of the nanoparticles. Further evidence that the desired MCTN was formed came from the weight percentages of the elements in the sample, which were determined to be Ti (27.4%), O (48.3%), and C (24.2%).

The anti-inflammatory effect of MCTN synthesized from *M. fragrans* extract was tested utilizing the BSA denaturation assay. The outcomes demonstrated that the dose-dependent inhibition of BSA denaturation by the MCTN demonstrated an excellent anti-inflammatory effect, which correlated with the previous report by Chahardoli et al. [31]. The highest concentration of MCTN (200 g/mL), which was discovered to be comparable to that of the positive control, diclofenac sodium, produced the greatest amount of inhibition. Curcumin, which is well known for its significant anti-inflammatory characteristics, may cause the anti-inflammatory activity of MCTN. Furthermore, an in vitro hemolytic assay was performed to evaluate the potential of M-TiO_2_ NPs and MCTN to induce hemolysis. MCTN showed higher hemolysis when compared with M-TiO_2_ NPs, although it remained below 5% lysis. The assay results revealed that the nanoparticles did not cause significant hemolysis of red blood cells at the various concentrations tested, which correlated with prior research outcomes by Chahardoli et al. [31]. This indicates that the nanoparticles are not detrimental to red blood cells and may possess good biocompatibility.

The study has examined the potential use of MCTN as an approach to addressing inflammation, with promising results in laboratory-based investigations. However, the transition from controlled experimental conditions to practical, real-world contexts necessitates further exploration. While our current focus has primarily been on short-term effects, there is an obvious need to investigate the long-term implications, including considerations of potential bioaccumulation. Although hemolytic activity has been assessed, a broader cytocompatibility evaluation that addresses inadvertent effects, nanoparticle dosage considerations, and coating stability must be analyzed as part of the future research trajectory. Lastly, a comprehensive examination of nanoparticle interactions within oral environments, coupled with adherence to regulatory standards, presents an avenue for prospective research in the field of dental sciences.

## Conclusions

This study represents a significant advancement by promoting the environmentally friendly creation of M-TiO_2_ NPs and characterizing them through techniques like UV spectrophotometry, FTIR, SEM, and EDAX. Importantly, the hemolytic assay has confirmed the compatibility of these nanoparticles, suggesting their potential as well-tolerated interventions. The findings highlight the effectiveness of TiO_2_ NPs derived from plant extracts, emphasized by their notable compatibility and low potential for cell damage. This research offers a promising avenue for the targeted management of oral inflammation in dental therapeutic applications, expanding the range of tools available for precise and effective treatments.
